# Knowledge of Learning Strategies and Motivation to Use Them: Similarities and Differences between School Levels

**DOI:** 10.3390/bs14100889

**Published:** 2024-10-01

**Authors:** Eve Kikas, Ita Puusepp, Mikk Granström, Kaja Mädamürk

**Affiliations:** 1 School of Natural Sciences and Health, Tallinn University, 10120 Tallinn, Estonia; kajamd@tlu.ee; 2 School of Educational Sciences, Tallinn University, 10120 Tallinn, Estonia; mikkg@tlu.ee; 3 Faculty of Educational Sciences, University of Helsinki, 00170 Helsinki, Finland; ita.puusepp@helsinki.fi

**Keywords:** learning strategies, strategy motivation, network analysis, middle school, high school

## Abstract

Students’ motivation to learn and the strategies they use during learning are two important factors that affect their learning outcomes. Some learning strategies are more effective, and some are less effective (deep and surface strategies). Both the knowledge of and motivation to use certain learning strategies depend on students’ individual characteristics (prior knowledge) and the context (classroom practices). The aims of the present study were (1) to examine differences between middle and high school students’ perceptions of the effectiveness of specific learning strategies and motivation to use effective strategies and (2) to explore relations between motivation, learning strategies, and grades in these two subgroups. The participants were 6287 middle school students (aged 13–16) and 5476 high school students (aged 17–20 years). The students completed an electronic questionnaire during school hours. Their perceptions of the effectiveness of specific learning strategies in certain learning scenarios and motivational beliefs (self-efficacy, utility, and cost) concerning the use of effective strategies were assessed with rating scales; additionally, students’ self-reported math and history grades were used. A network analysis was used to examine the relations between constructs. The results show small between-group differences in accordance with the theoretical expectations. The findings are discussed in relation to school level and educational context.

## 1. Introduction

The importance of self-regulation and the need to support the development of self-regulated learners is widely acknowledged [1,2]. An important aspect of self-regulated learning is the knowledge and application of learning strategies (LSs), i.e., the activities carried out during learning [3,4]. It is valuable for students to know the advantages and disadvantages of different LSs in specific learning situations, and thus, to possess good metacognitive knowledge of LSs [5]. Besides their knowledge of LSs, student’s self-regulatory choices and learning are influenced by motivational factors [1,6]. An influential theoretical framework for studying school-related motivation is the situated expectancy–value theory that differentiates two types of motivational beliefs: expectancies and values [7,8]. While a plethora of studies have shown the importance of subject-specific expectancies and values in learning and learning outcomes in various subject fields [7], learning-related strategy motivation [9] is far less studied [10,11]. However, as strategy motivation is critical for learning complex topics that presumes the use of deep LSs [10,12], more studies focusing on different school levels and subject domains are needed.

The aims of the present study were (1) to examine differences between middle and high school students’ perceived effectiveness of LSs and their motivation to use effective LSs and (2) to explore the relations among these constructs and math and history grades in middle and high school. We focused on middle and high school where successful learning presumes good knowledge of LSs and high motivation to use deep LSs. Math and history grades were used as indicators of achievement in subject domains that possibly differ in the specific LSs required for success. 

### 1.1. Learning Strategies and Their Effectiveness

Learning strategies (LSs) may be defined as a form of procedural knowledge or as goal-directed procedures that are intentionally and effortfully applied to support the regulation, execution, or evaluation of a particular learning problem or task [3]. With time, strategy usage becomes automated and is referred to as a skill [3]. Nonetheless, the use and differentiation of these two terms is not consistent among all researchers [4,13]. When put into practice, strategies and skills are the actual processes that students employ during learning, which directly influence both the process and outcomes of learning, being better predictors of learning outcomes than cognitive abilities [3,4]. 

LSs have broadly been categorized as supportive of deep or surface learning. Surface strategies focus on understanding or solving a problem, while deep strategies aim at integrating and transforming [3,13]. When using surface LSs, a student perceives new information and tends to mechanically repeat or memorize this information without attempting to integrate it with their prior knowledge [13,14]. With the use of deep LSs, a student not only perceives but also elaborates on new information, differentiates between more and less important information, and actively tries to integrate the new knowledge with their prior knowledge, resulting in the construction of new knowledge that is not simply a restatement of the new material [3]. It is well known that the use of deep LSs—compared to surface LSs—tends to support the comprehension of new material in a way that it can later be recalled and flexibly used when solving novel tasks [3]. The learning of complex topics is especially enhanced by the use of deep LSs rather than surface LSs [15,16].

However, these two types of strategies rarely form a clear-cut opposition due to differences in learner characteristics, tasks, and contexts [13]. The strategies each individual student uses differ across contexts and tasks [17], and each learning task may be successfully solved with the use of several different strategies [3]. Furthermore, each LS can be applied more or less effectively, which is related to deep or surface learning, respectively [18]. While some strategies are subject-specific (e.g., calculation strategies in math), others (e.g., self-testing) are more domain-general, i.e., useful across a variety of subject domains [13]. Still, the context matters, and the effectiveness of the same strategy may differ between domains—a strategy may be a highly adaptive or an optimal strategy in one domain but relatively less effective in another domain [3]. Also, the learner’s prior subject-specific knowledge influences the efficiency of using specific LSs [3]. Importantly though, being a successful self-regulated learner requires metacognitive knowledge of learning strategies and skills to apply them adaptively. Such knowledge and skills develop as students mature and learn. 

Primary school children use easily applicable surface LSs (rehearsal and rereading) that do not require well-structured prior knowledge and a high working memory capacity [19]. The use of such strategies may be sufficient for learning basic skills and factual knowledge but does not support learning and understanding complex material taught in subject lessons in higher grades. Although middle school students’ psychological processes—that have matured and developed during learning—allow the use of more complex and effective LSs that support deep learning [20,21], students tend to value and use surface LSs even at the end of middle school and later [16,22,23]. Still, cross-sectional studies have indicated that grade 9 students value and report using deep LSs more than younger students [22], and high school students tend to value deep LSs more than middle school students [24]. 

Students may discover the advantages of deep LSs independently when solving complex learning tasks and obtain information about learning from different sources like the internet and their parents. This may be more likely to happen during high school studies when students are required to comprehend and memorize large amounts of material and carry out homework projects, where successful completion depends on the context-specific application of different LSs. Also, high school students, compared with primary and middle school students, tend to have better prior knowledge, reasoning skills, and working memory capacity. Still, to support the development of metacognitive knowledge of LSs and a systematic adaptive use of LSs, explicit teaching and discussions about LSs are needed [25,26,27]. While teaching domain-general LSs, it is also critical to practice strategies for solving different tasks and explain why certain LSs are more adaptive in specific contexts [26,28]. However, authentic classroom observations have shown that teachers rarely explicitly talk about learning processes and the advantages of specific LSs [29,30]. Accordingly, in their study among grade 8 and 9 students, Olop et al. [31] found that only half of the participants were able to give examples of teachers’ recommendations for preparing for complex exams. Low emphasis on the explicit teaching of LSs may be one of the reasons for the lower knowledge and rare application of deep LSs in middle school students. 

### 1.2. Learning Strategies Addressed in This Study

We focused on three widely used deep strategies (distributing learning, retrieval, and integrating new knowledge with prior knowledge) and three surface strategies (massing, rereading, and highlighting). Nonetheless, as explained earlier, in certain situations, surface strategies may be more effective, and deep strategies may be less effective in terms of learning outcomes. In the following paragraphs, we describe the findings of empirical studies on the efficiency of these specific LSs and students’ knowledge and use of these strategies. 

Learning complex topics does not happen in a single shot, but rather, for deep learning, learners must repeatedly return to previously studied information. As such, distributing learning over time (also known as spacing) promotes better long-term retention than massing [32,33,34,35]. The advantages of distributing over massing have been shown both in experimental studies and educational settings, but its efficiency depends on the interval between study sessions [35,36,37]. In contrast, studies have indicated that students tend to believe that massing leads to better learning and test results than distributing [23]. Wiseheart et al. [35] argued that this may be true as massing supports short-term retention, which is often assessed in traditional education. Additionally, massing creates an illusion of learning, while, in contrast, the beneficial effect of distributing learning becomes visible after a longer period [38]. Differently from these studies, Granström and Kikas [24] found that both middle and high school students tended to perceive distributing learning for a test coming up in a few weeks to be more effective than studying intensively before the test. 

Retrieval, i.e., testing what has been learned, for example, by answering questions or generating explanations, has been demonstrated to be an effective LS (also referred to as self-testing, testing effect, and practice testing [32,39,40,41]). Retrieval has been shown to consolidate learned material, improve recall, and enhance meaningful learning at all ages and for learners of different ability levels [41,42]. Studies have also indicated that students value self-testing quite highly [24]. 

Rereading refers to reading a text or its parts repeatedly—something that may be carried out quite mechanically [33]. Although people can learn more from a second reading compared to the first reading, these gains tend to be small [43], and rereading is a much less efficient LS compared to those that require the learner to be more actively involved, for instance, retrieval [44,45]. Rereading has been shown to be a commonly reported study strategy [44]. Some studies that have manipulated retrieval practice versus rereading have found that students underestimate the benefits of retrieval practice and perceive rereading to be a more effective strategy [45], while others have found that students value retrieval more than rereading [24].

The essence of deep LSs is to actively recall what is known and to integrate new knowledge with prior knowledge. As such, creating verbal associations is a highly effective deep LS [46]. When new information is integrated with existing knowledge, it can be more easily applied in new contexts [5]. However, the efficiency of this LS also depends on what the student already knows [3], and thus, for each student, this strategy may be more effective in some domains (where the student possesses correct and well-structured prior knowledge) but less effective or even non-effective in other domains (where the student has low knowledge or misconceptions). 

Underlining or highlighting refers to marking specific parts of the text. This LS has the potential to benefit learning in two ways: (1) selecting what is important in the text elicits elaborative thinking (generative function), and (2) underlining or highlighting important parts makes it easier to identify them later (storage function [18]). However, students’ ability to use this strategy in an effective way varies a lot, and they often highlight/underline without really engaging in the selection of important information and thus use this strategy as a surface strategy [18]. Empirical studies have shown both the advantages and disadvantages of highlighting [33,47]. Students who highlight may later process the highlighted parts only, which makes reading more fluent and thus creates the illusion of learning [47]. Marking the text is a widely valued and practiced LS [18,24,47]. Still, Granström and Kikas [24] also found that high school students valued underlining less than more active LSs like creating associations. 

### 1.3. Learning-Related Motivational Beliefs and Their Relations to Learning

Motivational factors influence students’ self-regulatory choices and learning [1,6]. Intensive research has been carried out in the situated expectancy–value theoretical (SEVT) framework that describes two types of motivational beliefs: expectancies and values [7,8]. Students’ expectancies—their beliefs about how well they will perform in future tasks in a specific field—are closely related to self-efficacy [48]. Attainment value is the perceived personal importance of achievement and is related to one’s identity [7]. Intrinsic value refers to interest or inherent enjoyment gained from a given task. Utility value is the perceived relevance or usefulness of a given task or subject area regarding students’ current or future plans. Cost has been conceptualized as the time and energy that one must give up in order to engage in a given task, but it also involves the negative emotional or psychological consequences of this engagement [49] and has also been conceptualized as a separate construct from values [50,51]. Value components are interrelated and, in turn, related to expectancy or self-efficacy [7]. 

Motivational beliefs may support learning and outcomes via two pathways: the quantity and the quality of learning [52]. Learning quantity can be measured via persistence, frequency, intensity of study, etc. Learning quality refers to the use of more adaptive and deep LSs in specific learning situations. Since deep LSs are effortful and time-consuming, students who value certain tasks or domains (e.g., have higher interest in and see their usefulness in their lives) are more likely to employ these strategies. Previous research has shown that students with higher subject-related expectancies (academic self-efficacy) and subject-related task values tend to show higher task-persistent learning behavior and greater value and use of deep LSs [10,53,54,55]. Recently, researchers have started to examine the motivational beliefs related to learning, LSs, and their application. Namely, Karabenick et al. [9] differentiated between subject- or outcome-related and learning- or strategy-related motivation. Outcome-related motivational beliefs refer to motivation to study a specific topic, while strategy-related motivation (further strategy motivation) in an educational context refers to the motivation to use learning strategies. They emphasized that students decide to use a given strategy when they see its value and feel confident to use it in the specific learning situation. Regarding the cost component, since the use of deep LSs requires increased cognitive effort—including attention, working memory capacity, and higher reasoning skills [19,56,57]—the cost of using these strategies may also be perceived to be higher than that of applying surface LSs. However, students may still use deep LSs if they perceive the effectiveness of these strategies to be high, i.e., if they perceive the utility value of deep LSs to be high. Karabenick et al. [9] examined ninth-grade students’ LS-related utility and cost and the use of LSs in math lessons and found that, for most students, the strategies most often used were also the ones considered to be the most useful. However, reported LS use was not consistently related to perceived cost—for some students, a higher cost was linked to less frequent use of the LS, for some, cost and strategy use were relatively unrelated, and for others, the relations were even positive. 

As to changes in students’ motivation during school years, in most countries, subject motivation has been shown to clearly decline throughout middle school [7,58]. However, there is individual variability in such changes in motivation: person-oriented studies have described different trajectories of motivation, showing that the decline is more apparent for some students than for others [59,60]. So far, there are no studies on strategy motivation differences between middle and high school. Thus, our study aims to contribute to filling this gap. 

### 1.4. Network Approach to Examining Interrelations between Motivational Beliefs, LSs, and Achievement

Psychometric network models allow for the interrelations between a group of variables to be examined so that the relationship between any two variables is conditioned on all other variables in the system [61]. As such, this approach allows for a deeper understanding of the relationships between variables as a holistic and complex system. A network comprises nodes (variables) and edges between the nodes—namely, the associations between any two variables, when accounting for all other variables in the network, in contrast to bivariate correlations that account only for the relationship between any two variables. Although rather new in educational psychology research, this approach has recently been utilized in the SEVT framework to gain a better understanding of the interrelations between the components of motivational beliefs and achievement.

In one such study, Tang et al. [62] examined networks of multiple components of subject-specific motivational beliefs (expectancies, intrinsic value, attainment value, utility value, and cost) and academic achievement among Finnish and German sixth- to ninth-grade students in math and languages. By comparing the networks of different grade levels, subject domains, and countries, they showed differences as well as similarities in the interrelations of motivational beliefs and achievement across these networks. More specifically, concerning the similarities, across all of the networks, achievement had the strongest connection with expectancies and only weak associations with interest, utility, and attainment values. In another study, Lee et al. [63] used networks to examine the interrelations between math achievement and motivational beliefs concerning math among ninth-grade students. They found that expectancy was strongly connected to interest value, while it was weakly linked to utility value. They also found only expectancy to be connected to achievement.

While the network approach has been used to examine the interrelatedness of motivational constructs and achievement as a holistic system, to our knowledge, such an approach to study the interrelations between strategy motivation, perceptions of multiple specific LSs, and achievement as a holistic system has not yet been used.

### 1.5. The Cultural–Educational Background of This Study 

The current study was carried out in Estonia, a small country that was part of the Soviet Union from 1940 to 1991 and has since undergone rapid social changes. In Estonia, compulsory formal education starts at the age of seven and lasts for nine grades. During the first three years (in some schools, up to six years), the core subjects (i.e., math, literature, language, and science) are taught by the same class teacher. Later, different subjects are taught by different subject teachers. After grade 9, students can decide whether they want to continue their academic studies in high school (gymnasium) or in vocational school or whether they want to opt out of further academic studies. 

The aims and requirements of education are specified in the national curricula [64,65]. Besides the demands regarding academic skills, the curricula describe general (key) competencies and emphasize the importance of supporting the development of these competencies in all subjects. Learning to learn is one of the key competences included in educational policy documents as a main aim of learning to be developed in school [66,67,68]. Knowledge of learning—including knowledge of adaptive LSs—, skills to adaptively use LSs, and adaptive motivational beliefs form vital dimensions of learning to learn and thus should, according to curricular demands, be supported in all subjects in school. 

### 1.6. Aims

The aim of the current study was to examine the similarities and differences between middle and high school students’ perceived effectiveness of specific LSs and strategy motivation and the interrelations among these constructs and math and history grades. Middle and high school students’ learning differs both due to curricular demands (e.g., more complex, substantial, and integrated topics, but also more voluntary subject choices in high school) and learning procedures (e.g., longer periods devoted to learning specific subjects and longer lessons). Moreover, while middle school studies are obligatory, high school studies are voluntary, and thus, high school students may be more engaged in learning. The following research questions were used: 

First, how do middle and high school students differ in their perceptions of the effectiveness of deep (distributing, retrieval, and integrating) and surface LSs (massing, rereading, and highlighting)? 

Second, how do middle and high school students differ in their motivational beliefs regarding LSs?

Third, how are students’ perceived effectiveness of six LSs, their motivational beliefs, and their math and history grades interconnected? Are there differences in these associations between middle and high school students?

## 2. Materials and Methods

### 2.1. Subjects and Procedure

The participants of the study included 6287 middle school students (aged 13–16 years) and 5476 high school students (aged 17–20 years). Of the middle school students, 50.6% (3184) were girls, 46.0% (2891) were boys, and 3.4% (211) did not wish to identify their gender. In high school, 56.1% (3074) were girls, 41.3% (2260) were boys, and 2.6% (142) students did not want to identify their gender. The distribution of participants by grade was as follows: 2135 students were in 7th grade, 2128 in 8th grade, 2024 in 9th grade, 2303 in 10th grade, 1698 in 11th grade, and 1475 in 12th grade. 

First, approval from the Institutional Ethics Committee of Tallinn University, Estonia (application No. 6-5/43, decision No. 36 of the TLÜ Ethics Committee of 14 December 2023) was obtained. The survey was carried out between January and February 2024 by the Centre for Educational Innovation at Tallinn University, which conducts a nationwide student survey every two years. All schools in Estonia had the opportunity to participate in this research. Individual invitations with an enrolment form were sent to schools that had participated during previous years and to schools participating in different educational programs at Tallinn University. Other schools were able to register via the university website. Each school that registered for the survey received a unique link. The data were collected through the Qualtrics platform, which took 25–30 min. In total, students were asked to answer 6 different thematic blocks, including the topics focused on in this article. Participation in the survey was anonymous.

### 2.2. Measures

*Learning strategies.* To collect the data, scenario-based assessment questions were used. Students were asked to rate the effectiveness of six different learning strategies in the context of three learning scenarios [24,69,70]. Each learning scenario described a specific learning situation and one student who used a more effective LS and another student who used a less effective LS (for items of the used measures, see Appendix A). The three more effective LSs were distributing, retrieval, and knowledge integration, and the three less effective LSs were massing, rereading, and highlighting. Participants had to rate the effectiveness of the learning strategies regarding the specific learning situation on a 5-point evaluation scale (1—learning strategy is not very effective; 5—learning strategy is very effective). 

*Motivational beliefs questionnaire.* The questionnaire was developed based on the SEVT framework and assessed three motivational beliefs—self-efficacy (expectancy), perceived utility, and perceived cost of effective strategy use [7,10]. The questionnaire was presented after students had answered the LS questionnaire. The following instruction was given: “In the following, you will be presented with statements regarding the use of effective ways of learning. Effective ways of learning (also called learning strategies) are practices that help to understand, memorize, and later use the learnt material. For instance, it is more effective to plan learning and start learning for a complex test several weeks before the test. It is less effective to start learning the day before. Also, it is more effective to answer text-related questions, not to simply read the text several times. Evaluate to what extent the following statements apply to you. Use a scale of 1—strongly disagree … 5—strongly agree”. 

Initially, self-efficacy (e.g., “I know how to use effective ways of learning”) and perceived utility (e.g., “Using effective ways of learning helps me to understand the learning material better”) included three items, and perceived cost included six items (including effort, outcome, and emotional facets, e.g., “The use of effective ways of learning takes too much time”; “I don’t have the time to use effective ways of learning when I study at home because I have too many commitments”; “Using effective ways of learning is too exhausting for me”; for all items, see Appendix A). Confirmatory factor analysis was conducted to control the hypothesized loadings of the items on three factors. As one self-efficacy item did not load onto the presumed factor (Appendix B, Table A1), it was excluded, and there were two statements included in the self-efficacy factor (Appendix B). The internal reliabilities of the scales were between 0.587 and 0.844 (see Table A2 and Table A3), i.e., at least moderate (Cronbach’s α between 0.41 and 0.70 is moderate; α > 0.70 is high [71,72]).

In order to be able to compare selected constructs (self-efficacy, utility, and cost) of middle and high school students, a measurement invariance test was carried out. For this analysis, R version 4.2.1 with the Rstudio interface [73] and the statistical package lavaan ver 0.6–12 [74] were used. Since the χ2 test is sensitive to sample size [75], three additional fit indices were used to assess the goodness of fit of the model: the CFI, the RMSEA, and the SRMR. For CFI, values higher than 0.90 were considered appropriate [76]; for RMSEA, values lower than 0.80 were considered appropriate [77]; and for SRMR, values lower than 0.80 were considered appropriate [77]. The fit of the model was tested on groups of students in middle school and high school. Indicators for the middle school model were [χ2(6287) = 776.142, *p* < 0.001; CFI = 0.953, RMSEA = 0.061, SRMR = 0.045], and the model’s indicators were good. Indicators for the high school model were [χ2(5476) = 857.639, *p* < 0.001; CFI = 0.939, RMSEA = 0.069, SRMR = 0.049], and the model’s indicators were good.

Next, measurement invariance between the models of middle and high school groups was carried out. Measurement invariance analysis of data collected from middle school and high school students included similarity in factor structure (configural invariance), factor loadings (metric invariance), and free variables (scalar invariance). The ΔCFI, ΔRMSEA, and ΔSRMR values were estimated within the following ranges: >0.03 for weak invariance and results; <0.01 for strong invariance [75,78,79]. Since these fit indices stayed within the allowed ranges (Appendix C; Table A4), it can be concluded that the model structure is similar for both target groups and the groups can be compared. 

*Grades*. Students were asked to report their math and history grades from the last period. As the schools have different grading systems (e.g., some use a scale of A–F; some use a scale of 1–10), we rescaled the grades to match the scale of 2–5 that is used in the majority of schools. 

### 2.3. Data Analysis

To answer the first research question regarding the differences between middle and high school students in their perceptions of the effectiveness of six LSs as well as strategy motivation, Mann–Whitney U-test was used since, based on the Kolmogorov–Smirnov test, the data of both school levels did not follow normal distribution (*p* < 0.01). 

**Figure A1:**
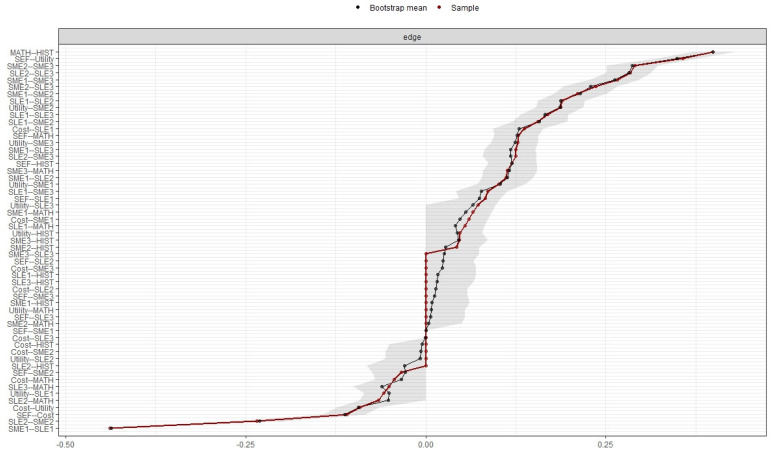
Non-parametric bootstrap (*n* = 5000) estimates and 95% confidence intervals (indicated by the grey area) for edge weights of middle school network.

**Figure A2:**
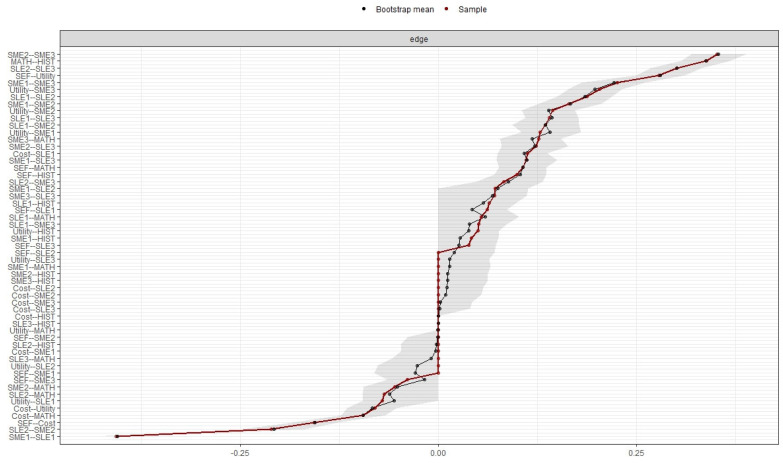
Non-parametric bootstrap (*n* = 5000) estimates and 95% confidence intervals (indicated by the grey area) for edge weights of high school network.

**Figure A3:**
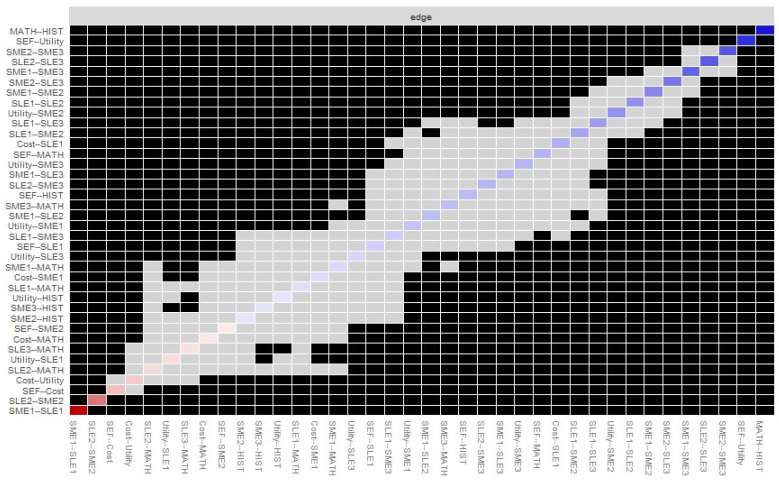
Bootstrapped difference tests (α = 0.05) between non-zero edge weights of estimated middle school network. Gray boxes indicate edges that do not differ significantly from one-another, while black boxes represent edges that do differ significantly from one-another. Colored boxes in the edge-weight plot correspond to the color of the edge in Figure 1.

**Figure A4:**
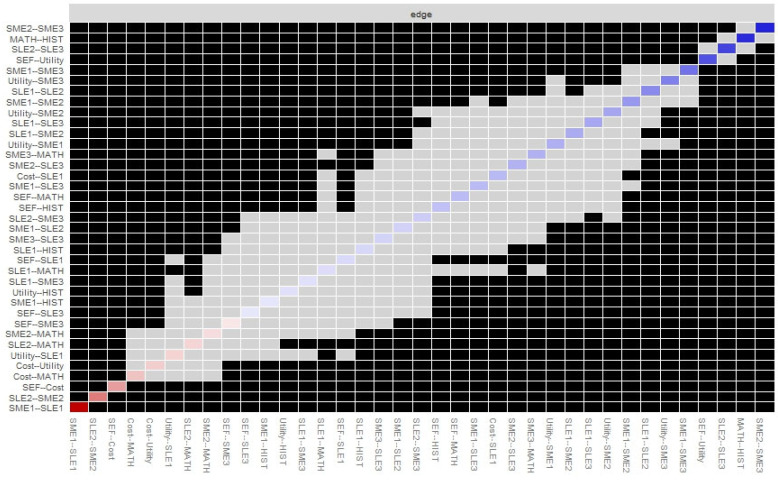
Bootstrapped difference tests (α = 0.05) between non-zero edge weights of estimated high school network. Gray boxes indicate edges that do not differ significantly from one-another, while black boxes represent edges that do differ significantly from one-another. Colored boxes in the edge-weight plot correspond to the color of the edge in Figure 1.

To answer the second research question and examine associations between students’ motivational beliefs about the use of LSs, how effective they perceive specific LSs to be, and their grades, a psychometric network analysis was used. Separate networks for middle and high school students were estimated. To estimate the networks, we used the *ggmModSelect* algorithm with stepwise estimation in the *bootnet* package [80] in R version 4.3.3 (with Rstudio version 2024.04.0). This estimation method searches for a model that fits the data best by iteratively adding and removing edges until the fit index of BIC is optimized. It has been shown to perform well in large sample sizes [81]. Due to the non-normality of the data, networks were estimated based on polychoric correlations [82]. To investigate the accuracy of the connections of the estimated networks, we conducted non-parametric bootstrapping with the *bootnet* package (see Figure A1 and Figure A2 in Appendix D for these results). Additionally, to enable the comparison of edge weights of the networks, the bootstrapped difference test with the *bootnet* package was used (see Figure A3 and Figure A4 in Appendix D for these results). 

Subsequently, the networks of middle and high school students were compared concerning the following aspects: (1) the overall network structure, (2) edge strength, and (3) global strength of the network. For this, the *NetworkComparisonTest* package was used [83]. When examining individual edge strength differences, Bonferroni–Holm correction for multiple comparisons was used. In order to handle missing data in the network analyses, multiple imputations with the *mice* package in R were utilized [84]. The missingness of data ranged from 0% for perceived effectiveness of specific LSs to 8.9% for history grades.

## 3. Results

### Differences in Level of Perceived Effectiveness of LSs and Strategy Motivation and Network Analysis

The results regarding the differences between middle and high school students (see Table 1) indicate that high school students tended to evaluate the strategies of distributing (z = −12.21), self-testing (z = −5.49), and integrating (z = −17.92) to be more effective than the middle school students; still, these evaluations were quite high at both school levels. The evaluations of the less effective strategies were quite similar between middle and high school. Regarding motivation, self-efficacy tended to be similar between the school levels, but utility (z = −11.99) as well as cost (z = −8.64) tended to be higher in high school. 

Bivariate correlations between all variables are presented separately for middle and high school in Table 2. The correlations are, overall, similar across the two school levels. While the bivariate correlations are not very high, they seem to be somewhat stronger in middle school.

**Figure 1:**
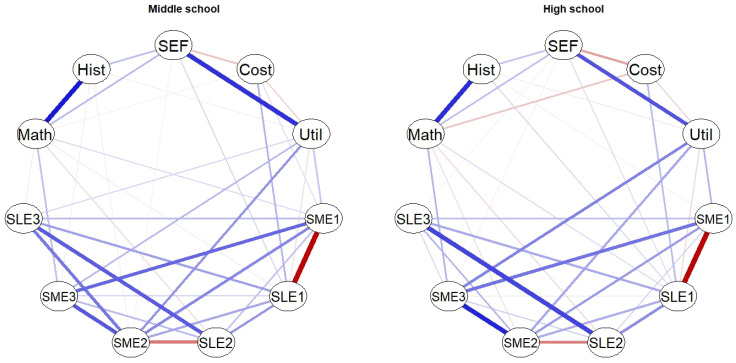
Estimated networks of motivational beliefs, perceived effectiveness of LSs, and achievement in middle and high school. Blue edges indicate positive connections and red edges indicate negative connections, with thicker edges referring to stronger connections. SME1—distributing; SME2—self-testing; SME3—knowledge integration; SLE1—massing; SLE2—rereading; SLE3—highlighting.

The estimated networks are presented in Figure 1. The networks indicate that math and history grades are strongly positively connected. Additionally, self-efficacy is strongly connected to utility, while it also has relatively weak connections with both grades. Utility value, on the other hand, is connected to the perceived effectiveness of more effective strategies. Otherwise, motivational beliefs are only weakly connected to perceptions of the different LSs, while overall, the interrelations between perceptions of specific strategies are stronger (see also Figure A3 and Figure A4 in Appendix D for the results of the bootstrapped difference tests of edge weights). More specifically, massing (SLE1) and distributing (SME1) show a strong negative connection. Furthermore, the more effective strategies are relatively strongly related to each other, with knowledge integration (SME3) being strongly connected to self-testing (SME2) and distributing (SME1). Also, the connection between the less effective strategies of highlighting (SLE3) and rereading (SLE2) is relatively strong. The only connection between perceptions of LSs and grades that, based on confidence intervals, differed from zero (see Figure A1 and Figure A2 in Appendix D) was the positive connection between math grade and knowledge integration (SME3).

To examine the differences in these networks between middle and high school, a network comparison test was conducted. The comparison test indicated that the middle school network had higher global strength (5.52) than the high school network (5.04; test statistic S = 0.48; *p* < 0.005). Additionally, the omnibus test of edge differences was significant (test statistic M = 0.11; *p* < 0.001). More specifically, there were marginal differences between middle and high school networks in the following edges: (1) the perceived effectiveness of highlighting and self-testing (test statistic E = 0.11; *p* = 0.055), (2) the self-efficacy and utility value (test statistic E = 0.08, *p* = 0.055), and (3) the utility value and perceived effectiveness of knowledge integration (test statistic E = 0.08; *p* = 0.055). As can be seen in Figure 1, the edge between perceptions of highlighting and self-testing as well as the edge between self-efficacy and utility value are marginally stronger in middle school, while the edge between the perceived effectiveness of knowledge integration and utility value is marginally stronger in high school.

## 4. Discussion

The present study aimed to examine the similarities and differences between middle and high school students’ perceived effectiveness of six LSs and their motivation to use effective LSs and to explore the relations among these constructs and math and history grades. We found that high school students tended to perceive the effectiveness of more effective strategies to be higher than middle school students, while there were no differences in the students’ perceptions of less effective strategies. Some differences in the motivation to use effective LSs also emerged. While the middle and high school students did not differ in self-efficacy when using effective LSs, the high school students perceived both the usefulness of effective LSs and the cost of applying them as higher compared to the middle school students. The relations among the perceived effectiveness of the six LSs, motivational beliefs, and grades were examined with a network analysis. While these constructs were more closely related to each other in middle school than in high school, their interrelations were overall similar across the two school levels.

### 4.1. Differences in Perceived Effectiveness of LSs and Motivational Beliefs between Middle and High School Students 

The students tended to evaluate the effectiveness of all LSs very highly, with the medians for less effective strategies being 3–4 and those for more effective strategies being even higher (4–5 on a scale ranging from 1 to 5). In line with an earlier study in Estonia [24], high school students tended to value more effective LSs higher than middle school students, while no differences were found for less effective LSs. More specifically, when asked about how to study (either a couple of days before the test or over three weeks) for a complex test to be completed after three weeks, the majority of students valued distributing over massing, and high school students tended to evaluate distributing as more effective compared to middle school students. Research has also indicated the efficiency of distributing over massing [32,33,34,35], although some studies have indicated that students value massing [23]. Also, self-testing (a highly effective strategy; see [32,39,40,41,42]) was the most highly evaluated strategy at both school levels (median 5), and high school students valued it even more than middle school students. Self-testing is easier to apply than some other effective strategies (e.g., integrating knowledge and composing drawings), especially as questions for self-testing are usually presented at the end of each chapter in textbooks in Estonia. The largest difference between middle and high school students emerged in the perceived effectiveness of integrating knowledge–thinking about what one knows about the topic and generating relations between newly learnt and already known knowledge. As the efficiency of this LS depends on the student’s current knowledge [3], the use of this strategy may be easier and more effective in high school. Moreover, due to their higher metacognitive awareness, high school students may be more capable of integrating and structuring material.

As to less effective LSs besides massing, rereading (reading twice) was valued less than the strategy presented as its alternative in the learning situation—self-testing. Students at both school levels evaluated rereading to be less effective similarly to an earlier study conducted among Estonian students [24]. The formulation of the application of this strategy (reading twice) refers to its use as a surface learning strategy [18,33,44,45]. In contrast, students evaluated highlighting to be quite effective. Earlier research has shown inconsistent findings on its efficiency [33,47] as its efficiency highly depends on whether students take time to think about what parts should be highlighted and whether they make additional comments [18]. 

Evaluations of the effectiveness of LSs may be treated as indicators of knowledge of LSs, an important aspect of successful self-regulated learning. The demonstrated good knowledge of the majority of students may be related to the Estonian educational context. Specifically, according to the demands of the Estonian national curricula [64,65], learning skills should be developed in school as part of learning competence. Electronic tools for assessing learning skills [28,85] are freely available for all schools and teachers, with in-service training courses held for teachers [86]. Additionally, separate learning competence lessons are provided in some schools. Earlier studies have also referred to Estonian teachers’ quite good knowledge of LSs [24,70,87]. Although observational studies have shown that teachers rarely talk about LSs explicitly in regular subject lessons, and a prior study indicated that only a minority of students can recall teachers’ advice on how to study for a complex test [31], teachers still use LSs in their teaching (i.e., model their usage; [29]). For instance, they give examples from their own lives, refer to the earlier learnt material, draw figures, and use models. Students observe these examples and may derive the usefulness of such strategies, even if they do not understand the reasons behind their efficiency. Given the complexity of learning tasks in high school, the effectiveness of deep LSs may become more evident through teachers’ demonstrated use of these strategies in the high school classroom compared to middle school. It should be emphasized that we asked students to evaluate the effectiveness of LSs with contrasting efficiency (used by two students), but we did not ask the participants to justify their answers, nor did we ask about which strategies they personally use. Even if students know that an LS is effective, they may not know how to use it.

The reason why the high school students tended to rank the more effective strategies higher may be related both to the learning context as well as the students’ better metacognitive skills. Specifically, in high school, learning tasks are more complex and time-consuming, and the amount of information students have to learn in different subjects is greater than in that middle school. Students’ knowledge is often assessed after having covered a broad range of material, and such assessments typically encompass more topics in high school than in middle school. Thus, students may need more time and strategies to integrate information, and therefore, they might discover the advantages of some LSs on their own, even without explicit teaching [3,15]. Using complex LSs is also easier due to their better subject-specific knowledge that enables them to integrate learning material with prior knowledge [3].

Differently from many earlier studies that have examined motivation and changes in motivation to learn specific subjects [7,54,58], we studied the motivation (self-efficacy, perceived utility, and cost) to use effective learning strategies [9]. Both the middle and high school students reported a similar level of self-efficacy in using effective LSs (median 3). In contrast, both the perceived utility and cost of effective LSs was higher in high school. As described before, the students rated the effectiveness of deep LSs higher in high school; therefore, the finding that the utility of effective LSs is perceived to be higher in high school may be related to students’ more frequent use of these LSs due to complex learning tasks. When justifying their finding that the reported use of LSs was not consistently related to the perceived cost, Karabenick et al. [9] suggested that students may have low metacognitive knowledge of strategy use. They also proposed that while students may know how useful an LS is, they may not be aware of its cost. Here, also, high school students may have higher metacognitive awareness and a better understanding of what using deep LSs entails (effort, time, reasoning, challenges working memory, etc.) and thus perceive their cost as higher. It is also possible that middle and high school students (even each individual student) may differently conceptualize what an effective LS is.

### 4.2. Associations between Perceived Effectiveness of LSs, Strategy Motivation, and Grades

Overall, the interrelations between evaluations of the studied LSs, motivational beliefs regarding strategy use, and grades were similar across the two school levels. In order to memorize and understand new knowledge, i.e., to learn successfully, a student needs to know the advantages and challenges of different LSs, but also to have the skills to choose and use the most effective strategies regarding the particular context and task [3,5,13]. The network analyses showed quite similar relations among the students’ evaluations of LSs—there were generally positive relations among (1) more effective LSs and (2) less effective strategies, and there were (3) some negative relations between pairs of more and less effective strategies for a particular learning scenario (Figure 1). This means that students who tended to perceive, for example, knowledge integration as an effective strategy tended to also evaluate self-testing and distributing as effective strategies. Acknowledging the efficacy of these three LSs gives a good starting point to effective learning and becoming a successful self-regulated learner. In contrast, students who value these strategies lowly may struggle with learning. Additionally, evaluations of the less effective strategies were all positively connected to each other, with the connection between rereading and highlighting being especially strong. It is possible that while studying, students who value highlighting as an effective LS may later reread and process the highlighted parts of the material only. Therefore, while highlighting has the potential to benefit learning if it is used to elicit elaborative thinking and to identify important parts of the learning material [18], the strong positive connection between highlighting and rereading in this study might indicate that students tend to use highlighting as a surface strategy [33,47]. Using these strategies together with massing may be especially problematic in terms of long-term retention but may lead to success when short-term retention is tested [35,38]. As students had to evaluate the efficiency of pairs of more and less effective LSs, negative relations between these pairs could be expected. Especially strong negative relations were found between massing and distributing as well as between self-testing and rereading. Interestingly though, there was a nearly non-existent connection between evaluations of knowledge integration and highlighting; this might be due to inconsistent relations between the evaluations of these two strategies across students (possibly groups of students evaluating both highly as well as groups evaluating one to be effective and the other as not effective). Nonetheless, we did not study the students’ use of the specific strategies, but only their perceived effectiveness of these strategies in specific learning situations.

Regarding motivational beliefs, self-efficacy in using effective LSs and the perceived utility of effective LSs were strongly positively connected to each other, while their negative relations with the perceived cost of effective LSs were weaker. It is possible that students who perceive the use of effective strategies as more useful have better metacognitive knowledge of strategy use, and therefore, they also feel more capable of successfully applying effective LSs. In contrast, it is possible that the connections with cost are weak as even when students feel capable of using effective LSs and see the utility of their use, they may simultaneously also perceive their use to be effortful and time-consuming.

While the higher perceived utility of effective LSs was positively connected to evaluating more effective LSs highly, SEF and cost had only a few very weak connections with evaluations of specific LSs. It is probable that students who have better metacognitive knowledge of LSs, which is reflected in their evaluations of deep LSs as more effective, also perceive the use of effective strategies to be more useful. In contrast, perceiving the use of effective LSs as more or less costly and perceiving oneself as more or less capable of using effective LSs are not necessarily related to how effective one perceives a specific LS to be. It is possible that students have low metacognitive knowledge of strategy use—meaning they are not well aware of the cost of effective LSs—and therefore, no strong connections with perceived cost and evaluations of specific LSs emerged [9]. Cost was nevertheless positively but relatively weakly connected to the evaluation of one less effective LS: massing. As described above, in general, students in this study tended to value distributing over massing when studying for a complex test. Nonetheless, there were also students who perceived massing to be effective in such a situation. Students reporting the cost of using effective LSs to be high may additionally perceive the subject-related cost to be high and may thus perceive intense learning just before a test to be an effective strategy to pass the test. 

Despite having focused on grades as indicators of academic knowledge and success in two subjects that are as different as possible, the relations between math and history grades were strong. While self-efficacy in using effective LSs was positively, although weakly, connected to math and history grades, connections between grades and utility value and perceived cost were non-existent or nearly non-existent (based on the confidence intervals, the utility value did not have connections to grades, and a higher perceived cost was connected to lower math grades only in the high school network). Similarly, Tang et al. [62] and Lee et al. [63], when using a network approach, found expectancies to have the strongest connection to achievement, with value components showing weaker or non-existent links, but both of these studies focused on subject motivation and not strategy motivation. As to the associations of grades with the perceived effectiveness of the specific LSs, based on confidence intervals, only the connection between math grades and knowledge integration differed from zero, with higher math grades being weakly connected to greater perceived effectiveness of knowledge integration. Since in math, new topics usually require integrating prior knowledge, this type of connection is expected. Although we examined LSs that are considered general and applicable to all learning, these—albeit small—differences in relations refer to the context specificity of LSs (see also [3]).

While overall, the interrelations between evaluations of LSs, strategy motivation, and grades were similar across the two school levels, the global strength of the middle school network was significantly greater, which indicates that when studying the associations between these constructs holistically, they were more closely related with each other in middle school than in high school. It is possible that by high school, students have higher metacognitive knowledge regarding LSs (as also indicated by their higher evaluations of effective LSs in the present study), and as they learn to value the effectiveness of deep LSs more, these evaluations are not as connected to their evaluations of less effective or surface LSs. For example, middle school students who valued self-testing as an effective strategy tended to give a similarly high evaluation to the strategy of highlighting. This positive connection between self-testing and highlighting, though, was marginally weaker in high school. In addition to this marginal difference, there were a few other differences regarding the individual edges of the two networks, but these were only marginal as well. 

## 5. Limitations, Conclusions, and Future Directions 

When considering the present findings, it also important to take into account the several limitations of this study. First, it was not a longitudinal study, and therefore, we can only talk about differences between middle and high school students. In the future, longitudinal studies should be carried out to examine the development of the knowledge of LSs and strategy motivation. Second, participation in the study was voluntary for school, and thus, only schools who pay greater attention to developing students’ nonacademic characteristics, including their learning competence, might have participated. Thus, we cannot generalize the findings to all Estonian schools. Third, although we explained what is meant by effective LSs when assessing the students’ strategy motivations, it may still be that younger and older students interpreted the meaning of effective LSs differently. Further studies with different methods (e.g., interviews) are needed to learn about students’ interpretations. Fourth, grades are not the best indicators of knowledge and skills as they are teachers’ subjective assessments, and grading scales vary between schools. Also, we used self-reports that do not capture all aspects of assessed motivational constructs, and students might not interpret the statements and rating scales in a similar way [88]. 

In general, the students evaluated the effectiveness of all LSs quite highly. Still, they tended to evaluate the effectiveness of more effective LSs higher than that of less effective strategies, and the high school students valued more effective LSs more compared to the middle school students. However, as we noted before, we assessed the students’ evaluations on rating scales. We did not ask the students to justify their answers, and we do not know if their existing knowledge is applied in classroom learning [31,89]. Therefore, the present findings provide a good starting point for further teaching about learning and LSs and also for further studies. Researchers emphasize the importance of metacognitive knowledge of learning and LSs [5,25,26], and only more detailed studies (e.g., with interviews) can tap into this knowledge. In the future, it would also be important to examine whether and how these general LSs are used in different learning contexts (e.g., different subjects) by students with different prior knowledge, cognitive abilities, and motivation. Also, what really matters for learning is what kind of LSs are used and how they are used. Further studies (e.g., observations and case studies) are needed to examine actual classroom practices and their efficiency. 

Additionally, in the present study, we studied students’ motivation to use effective LSs in general and the associations of these general strategy-related motivational beliefs with the perceptions of specific LSs and grades. In the future, it would be fruitful to examine interrelations with motivational beliefs regarding each of the studied LSs separately. Furthermore, longitudinal designs using more intensive data collection would enable examining not only between-individual differences, but also within-individual differences in the interrelations between strategy motivation, perceptions or use of LSs, and achievement as well as temporal associations, which could reveal important nuances regarding these interrelations. 

Overall, the present findings indicate some differences between middle and high school students’ strategy motivation and perceived effectiveness of specific LSs. Additionally, by using a network approach, this study contributes to our understanding of the interconnectedness of students’ perceptions of the effectiveness of multiple specific more and less effective LSs, strategy motivation, and achievement.

## Figures and Tables

**Table 1:** Differences in the level of perceived effectiveness of LSs and strategy motivation (Mann–Whitney U-test).

	Middle School					High School						
	N	Min–Max	Mdn	M	SD	N	Min–Max	Mdn	M	SD	U	z
Distributing	6111	1–5	4.00	3.80	1.08	5331	1–5	4.00	4.04	0.99	14,238,480.00	−12.21 *
Self-testing	6111	1–5	5.00	4.25	0.95	5331	1–5	5.00	4.38	0.82	15,412,847.50	−5.49 *
Integrating	6111	1–5	4.00	3.91	0.92	5331	1–5	4.00	4.21	0.81	13,324,815.00	−17.92 *
Massing	6111	1–5	3.00	3.24	1.11	5331	1–5	3.00	3.21	1.04	16,031,128.50	−1.52
Rereading	6111	1–5	3.00	3.15	1.03	5331	1–5	3.00	3.12	1.02	16,029,479.50	−1.54
Highlighting	6111	1–5	4.00	3.90	0.88	5331	1–5	4.00	3.87	0.85	15,942,688.50	−2.10
Self-efficacy	6028	1–5	3.00	3.17	0.84	5249	1–5	3.00	3.16	0.81	15,727,984.50	−0.55
Utility	6028	1–5	3.67	3.72	0.83	5249	1–5	4.00	3.90	0.76	13,770,486.50	−11.99 *
Cost	6028	1–5	3.00	3.00	0.81	5249	1–5	3.20	3.13	0.80	14,335,881.50	−8.64 *
Math grade	5753	2–5	4.00	4.00	0.87	5028	2–5	4.00	3.85	0.83	12,912,123.50	−10.16 *
History grade	5736	2–5	4.00	4.32	0.78	4733	2–5	4.00	4.29	0.74	13,120,150.00	−3.22 *

* *p* < 0.01.

**Table 2:** Bivariate correlations (Spearman’s ρ) between the variables.

	1.	2.	3.	4.	5.	6.	7.	8.	9.	10.	11.
1. Distributing	-	0.25 *	0.33 *	−0.31 *	0.00	0.13 *	0.06 *	0.30 *	−0.08 *	0.05 *	0.04 *
2. Self-testing	0.31 *	-	0.39 *	0.02	−0.07 *	0.15 *	0.09 *	0.30 *	−0.02	0.03	0.05 *
3. Integrating	0.37 *	0.38 *	-	−0.02	0.05 *	0.19 *	0.11 *	0.35 *	−0.04 *	0.13 *	0.09 *
4. Massing	−0.30 *	0.06 *	0.03 *	-	0.18 *	0.14 *	0.03 *	−0.10 *	0.12 *	0.05 *	0.06 *
5. Rereading	0.06 *	−0.03 *	0.10 *	0.17 *	-	0.28 *	0.03 *	−0.05 *	0.04 *	−0.04 *	−0.01
6. Highlighting	0.19 *	0.26 *	0.23 *	0.17 *	0.27 *	-	0.07 *	0.11 *	0.01	0.00	0.02
7. Self-efficacy	0.12 *	0.14 *	0.19 *	0.05 *	0.04 *	0.10 *	-	0.33 *	−0.20 *	0.18 *	0.17 *
8. Utility	0.31 *	0.34 *	0.34 *	−0.05 *	0.01	0.19 *	0.41 *	-	−0.16 *	0.11 *	0.12 *
9. Cost	−0.03	−0.05 *	−0.01	0.11 *	0.05 *	0.01	−0.17 *	−0.17 *	-	−0.12 *	−0.06 *
10. Math grade	0.14 *	0.13 *	0.21 *	0.02	−0.04 *	0.01	0.25 *	0.20 *	−0.08 *	-	0.30 *
11. History grade	0.11 *	0.13 *	0.17 *	0.03	−0.04 *	0.05 *	0.23 *	0.20 *	−0.06 *	0.37 *	-

*Note*. The correlations of middle school data are presented below the diagonal and high school above the diagonal. * *p* < 0.01.

## Data Availability

The raw data supporting the conclusions of this article will be made available by the authors without undue reservation.

## Appendix A. The Items of the Used Measures

**Learning situations and pairs of more (a) and less effective (b) learning strategies**

1. Imagine two students who must study three pages of text from a textbook to prepare for a test. Both students have thoroughly read the text once. After that, they act differently.
  - Student B tests his/her knowledge. He/she closes the book and writes down everything he/she recalls about the read pages.
  - Student A reads the text once more.
2. Students have three weeks to prepare for a test. They must study a chapter from their textbook and be prepared to answer questions about it. Students A and B study for the same amount of time but use different learning strategies.
  - Student A divides studying and answering questions over the period of three weeks.
  - Student B studies and answer question intensively for one or two days before the test.
3. Students must familiarize themselves with a new topic before the lesson. For homework, they must read a chapter from the book. Their knowledge about the topic is not very good. Both students study the new topic for the same amount of time but use different strategies.
  - Student A reads the text through and then tries to make sense of what he/she read. He/she thinks about what he/she already knows about the topic and tries to integrate the new information with what he/she knows.
  - Students B reads the text thoroughly and marks important parts of the text.

**Motivational beliefs questionnaire**

Self-efficacy

1. I know how to use effective ways of learning.
2. I feel confident when using effective ways of learning.
3. Compared to my classmates, I am better at using effective ways of learning.

Cost

1. The use of effective ways of learning takes too much time.
2. Learning using effective ways of learning means that I have to give up some other activity that is important to me.
3. I don’t have the time to use effective ways of learning when I study at home because I have too many commitments.
4. I don’t have the time to use effective ways of learning when I study at school because there are too many tasks.
5. Using effective ways of learning is too exhausting for me.
6. Using effective ways of learning is too effortful for me.

Utility

1. Using effective ways of learning helps me to understand the learning material better.
2. Knowing how to learn effectively will help me to cope better in the future.
3. Using effective ways of learning helps me to be more successful in my studies.

## Appendix B. Confirmatory Factor Analysis Loadings and Uniqueness Values

**Table A1:** Factor loadings and uniqueness values. Original data and whole sample.

Indicator	1	2	3	Uniqueness
Self-efficacy 1		0.465		0.575
Self-efficacy 2			0.594	0.665
Self-efficacy 3			0.616	0.503
Cost 1	0.586			0.665
Cost 2	0.747			0.445
Cost 3	0.674			0.48
Cost 4	0.567			0.696
Cost 5	0.705			0.449
Cost 6	0.636			0.615
Utility 1		0.69		0.519
Utility 2		0.783		0.374
Utility 3		0.83		0.344

**Table A2:** Confirmatory Factor Analysis Factor Loadings in Middle School.

Factor	Indicator	Estimate	Std. Error	z-Value	*p*	Cronbach’s α
Self-efficacy	Sef 2	0.502	0.013	39.1	<0.001	0.584
	Sef 3	0.814	0.016	52.4	<0.001	
Cost	Cost 1	0.614	0.012	49.4	<0.001	0.806
	Cost 2	0.784	0.011	72.9	<0.001	
	Cost 3	0.756	0.011	71.8	<0.001	
	Cost 4	0.599	0.012	50.4	<0.001	
	Cost 5	0.793	0.011	74.1	<0.001	
	Cost 6	0.699	0.013	54.9	<0.001	
Utility	Utility 1	0.661	0.01	65.5	<0.001	0.801
	Utility 2	0.794	0.01	79.5	<0.001	
	Utility 3	0.799	0.01	81.4	<0.001	

**Table A3:** Confirmatory Factor Analysis Factor Loadings in High School.

Factor	Indicator	Estimate	Std. Error	z-Value	*p*	Cronbach’s α
Self-efficacy	Sef 2	0.525	0.017	31.7	<0.001	0.682
	Sef 3	0.764	0.02	38.6	<0.001	
Cost	Cost 1	0.583	0.015	38.3	<0.001	0.844
	Cost 2	0.765	0.013	60.3	<0.001	
	Cost 3	0.778	0.013	60.3	<0.001	
	Cost 4	0.515	0.015	35.1	<0.001	
	Cost 5	0.796	0.013	61.7	<0.001	
	Cost 6	0.68	0.015	44.6	<0.001	
Utility	Utility 1	0.578	0.011	50.9	<0.001	0.837
	Utility 2	0.723	0.011	63.8	<0.001	
	Utility 3	0.741	0.011	66.5	<0.001	

## Appendix C. Model Fit Indices and Invariance Testing Results

**Table A4:** Model fit indices and invariance testing results.

	χ2	df	*p*	CFI	RMSEA	90% CI	SRMS	∆χ2	∆df	*p*	∆CFI	∆RMSEA	∆SRMS
Configural Invariance	2242.833	164	<0.001	0.949	0.063	[0.061, 0.063]	0.043	-	-	-	-	-	-
Metric Invariance	2278.201	188	<0.001	0.948	0.059	[0.057, 0.061]	0.044	35.4	24	0.06	−0.001	−0.004	0.001
Scalar Invariance	2831.882	212	<0.001	0.935	0.062	[0.060, 0.065]	0.047	553.681	24	0	−0.013	0.003	0.003
